# Mercury sodium exospheric emission as a proxy for solar perturbations transit

**DOI:** 10.1038/s41598-018-19163-x

**Published:** 2018-01-17

**Authors:** Stefano Orsini, Valeria Mangano, Anna Milillo, Christina Plainaki, Alessandro Mura, Jim M. Raines, Elisabetta De Angelis, Rosanna Rispoli, Francesco Lazzarotto, Alessandro Aronica

**Affiliations:** 1 INAF-IAPS, Roma, Italy; 2 ASI, Roma, Italy; 3 University of Michigan, Ann Arbor, MI MI 48109, USA

## Abstract

The first evidence at Mercury of direct relation between ICME transit and Na exosphere dynamics is presented, suggesting that Na emission, observed from ground, could be a proxy of planetary space weather at Mercury. The link existing between the dayside exosphere Na patterns and the solar wind-magnetosphere-surface interactions is investigated. This goal is pursued by analyzing the Na intensity hourly images, as observed by the ground-based THEMIS solar telescope during 10 selected periods between 2012 and 2013 (with seeing, σ < = 2″), when also MESSENGER data were available. Frequently, two-peak patterns of variable intensity are observed, located at high latitudes in both hemispheres. Occasionally, Na signal is instead diffused above the sub-solar region. We compare these different patterns with the *in-situ* time profiles of proton fluxes and magnetic field data from MESSENGER. Among these 10 cases, only in one occasion the Na signal is diffused above the subsolar region, when the MESSENGER data detect the transit of two ICMEs. The selected cases suggest that the Na emission patterns are well related to the solar wind conditions at Mercury. Hence, the exospheric Na emission patterns, observed from ground, could be considered as a ‘natural monitor’ of solar disturbances when transiting near Mercury.

## Introduction

The Na bright doublet emission at 5890–96 Å, thanks to its good visibility also from the ground, is broadly used to study the exosphere of Mercury^1–6^. Since the discovery of Na component^7^, a large dataset of images were recorded in which both recurrent and variable patterns are observed. By means of the ground-based THEMIS solar telescope (^8^, and references therein), sequences of data for many hours per day are now collected and can be used to study the high dynamicity and morphology of the exosphere of Mercury through its Na component.

The dynamics of the exosphere of Mercury is due to the processes induced by environmental agents. Being that this planet is very close to the Sun, it is strongly exposed to solar thermal and UV radiation. Moreover, it is also heavily bombarded by micrometeoroids and by solar wind plasma, along with embedded Interplanetary Magnetic Field (IMF).

Mangano and coauthors^8^ showed that the exospheric Na ground-based observations quite often (61%) exhibit a two-peak pattern located at high latitudes in both hemispheres, with variable intensity, often persisting for several hours, or even days^8–10^. The peak area is approximately the cusps projection on the surface, thus supporting the idea that solar wind precipitation through the polar cusps plays a key role. The Na in the upper surface layer is released and then photo-ionized, and starts circulating and accelerating, as actually detected by MESSENGER/FIPS^11^.

The recurrent patterns observed by THEMIS in the Na exosphere of Mercury can be divided into two main categories: the already cited configuration of two separate peaks in the polar regions and, conversely, a diffused emission on the dayside, sometimes peaking in the subsolar region. Also asymmetries between the dawn/dusk terminator and the limb zones, or between dawn and dusk terminators, have been observed^2,12–14^. Moreover, a strong variability with true anomaly angle (TAA) of the global brightness intensity is now well known^5^. The most important cause of variation in intensity with TAA is the variation in g-value (i.e. photons/cm^2^/sec/atom). Other composite effects are induced by two factors: one related to the variability of the radiation pressure acceleration along the orbit of Mercury, and one related to the processes of deposition and migration of Na in the exchange between surface and exosphere.

Despite the large number of empirical and theoretical studies, the source processes of the Na exosphere of Mercury still remain debated. While the patterns and the variability observed from Earth point to a link with the plasma impacting the surface, the ion-sputtering process alone is not expected to be able to release enough Na to account for the intensity of the observed peaks at high-latitudes^15^. Furthermore, the mostly-equatorial measurements performed by the UV spectrometer MESSENGER/MASCS show a seasonal repeatability of the Na vertical profile^16^, consistent with a release mainly driven by the photon stimulated desorption (PSD)^13^. Moreover, MESSENGER/GRS discovered a higher Na abundance in the Northern hemisphere with respect to the equatorial regions^17^ that could affect the Na release at these latitudes. On the other hand, the Na surface abundance is limited and cannot sustain the observed emission rate indefinitely^18^ unless some refilling mechanism, or diffusion from deeper levels, is considered.

The apparent discrepancy of MESSENGER/MASCS observations with respect to ground-based observations is probably due to the fact that MASCS observations are taken from a different point of view allowing only detection of the limb exosphere at the equatorial region. In fact, data taken when the line of sight is close to the polar regions are presently under analysis^19^. As a result, the two-peak distributions are not detectable, and consequently the corresponding Na-release scenario observed from ground cannot be fully confirmed by *in-situ* MASCS observations. Following a suggestion by^12^, Leblanc and Johnson^5^ interpret the high-latitude Na emission coupled with a quasi-stable Na exosphere (varying only seasonally) as the result of deposition in the shadowed areas of Na pushed by radiation pressure toward the night-side. The slow rotation of the planet then brings this Na reservoir into sunlight, causing (via PSD) an enhanced release of Na at the dawn terminator, and a very slow varying Na exosphere. However, this prediction conflicts with the ground-based observations showing that the Na emission occurs mainly close to the noon meridian^8^, and that it can exhibit short-term intensity variations, with a timescale shorter than 1 hour^4,9^ and could go down to 10 minutes^10^.

In summary, particle precipitation seems to be the most reliable mechanism to justify the observed patterns. Since it is difficult to explain such features on the basis of a single release mechanism, Mura *et al*.^15^ suggested a synergy of more than a single process to account for the observational evidence of two-peak features, and, more generally, of features that do not show any simple dependence with the Sun illumination. Such a synergic scenario combines the precipitation of ions falling on the surface (e.g.: in the case of two-peak emission, from the cusp footprints) with the enhancement of the Na diffusion from inside the regolith grains. Laboratory measurements of PSD from regolith^20^ lead indeed to the estimation of the unconstrained flux, at the sub-solar point, of ~2 10^9^ cm^−2^ s^−1^ ^21^. This value should result in a tangential column density of almost 10^13^ cm^−2^, which is not observed. Once rescaled to match observations, surface fluxes should be close to 10^7^ cm^−2^ s^−1^. This is more likely to be sustainable by the regolith Na contents in case of diffusion from interior^22^, enhanced by the solar wind precipitation. Each precipitating proton has roughly 10% probability of freeing a deep, bonded Na particle, which is then rapidly emitted by PSD. Because of the intrinsic ballistic times of particles, of the photoionization lifetime and of the Jeans escape probability, the Na exosphere should exhibit variations on timescale that, depending on the TAA, are of the orders of one hour^22^.

If we assume that particle precipitation is one of the major processes causing Na surface release and consequent refilling of the exosphere, the IMF magnitude and direction should be important parameters for the location of the Na emissions. At Mercury, the average subsolar magnetopause standoff distance is ∼1.45 R_M_^23^. As it happens at the Earth, the open-closed field line boundaries of Mercury’s magnetosphere map to high latitude dayside regions defining magnetospheric cusps. As observed by MESSENGER, the cusp appears as a strong enhancement in plasma flux, composed of both solar wind and planetary ions^24–26^, standing between two regions of much lower plasma density and in a magnetic field depression, attributed to the diamagnetic influence of the plasma. In a statistical analysis, the cusp is a broad, highly variable region located around 56°–84° N magnetic latitude and 7–16 h local time^27^. Clear signatures of plasma precipitation in the northern cusp are evident in both MESSENGER plasma and magnetic field data in the vast majority of orbits that cross this region. In fact, Mercury’s dayside magnetopause is frequently experiencing reconnection as a result of the low Alfvénic Mach number (${{\rm{M}}}_{{\rm{A}}}={{\rm{V}}}_{{\rm{SW}}}{/V}_{{\rm{A}}}$) conditions, where V_SW_ is the solar wind bulk velocity and V_A_ is the Alfvén speed^28^. The latter is given by ${{\rm{V}}}_{{\rm{A}}}=B/\sqrt{{{\rm{\rho }}{\rm{\mu }}}_{0}}$, relating the magnitude of the magnetic field (B), the mass density of the plasma (ρ) and the permeability of free space (μ_0_). This induces a low plasma β in the magnetosheath (where β is the ratio of plasma thermal to magnetic pressure), associated with the planet’s location in the inner heliosphere. Massetti *et al*.^10^ indicate that, by taking into consideration the mean values of both solar wind speed and density at the orbit of Mercury (e.g.^29^), a value of IMF |B| > 25 nT is likely to be associated to a low M_A_ (<5) upstream. This condition causes higher reconnection rate at Mercury, nearly regardless of the IMF orientation.

Plasma precipitation usually occurs within open field line regions, like the Flux Transfer Events, which have been identified to occur as “FTE showers”^30^, depending on reconnection rates, very frequent at Mercury, resulting in a broad area of intense plasma precipitation on the surface, especially in case of solar wind disturbances. Occasionally, precipitation onto the surface is not only limited within open field lines regions. Plasma impact onto most of the dayside surface could occur especially in extreme conditions, like interplanetary coronal mass ejection (ICME) or high-speed streams (HSS), as predicted by models and inferred by MESSENGER observations^31–34^. The quasi-neutral-hybrid model^31^, considering symmetric dipolar internal magnetic field, showed that the magnetosheath plasma could be pushed very close to the surface of Mercury, near the sub-solar point. While during HSS the induction effects produce a temporarily increase of the Mercury’s magnetic moment^35^, the shielding is less efficient during ICME events of slow and dense plasma. In fact, the MESSENGER observations during extreme conditions^32^, showed that the ICMEs produce thick, low-β plasma depletion layers; hence, there is a relevant probability (30%) that the magnetopause plasma can impact onto the planet’s surface^33^.

## Data Analysis

Here we use a subset of data (years 2011–2013) of the hourly Na images obtained at the THEMIS solar telescope, together with the *in-situ* magnetic field measurements and proton spectrograms, respectively coming from the MAG and FIPS sensors onboard the MESSENGER spacecraft. For 10 non-contiguous days the Na emission was observed for a sequence of 3 or more hours (not necessarily consecutive) within each selected observation day, in good seeing conditions (σ <  = 2″). All of them, except for one case, exhibit double-peak patterns, almost constantly observed throughout the whole considered day. We focus on two representative cases of double-peak patterns (2012, June 7, and 2013, May 25), as well as on the only case presenting a different emission pattern, diffused on the dayside (2012, September 20). In Table 1, the three selected cases are listed with indication of time and seeing values. Images are corrected with tiptilt (see description section below) and, hence, the associated seeing values should be considered only as upper limits. It follows that main images features are reliable. The seeing errors refer to the atmospheric fluctuations occurrence during the ~60 min needed for each observation. In summary, we can list the average seeing for the three sequences, as well as the relative errors.

- (07/06/2012): Average seeing = 1.8 ± 0.7″.
- (25/05/2013): Average seeing = 1.7 ± 0.7″.
- (20/09/2012): Average seeing = 1.8 ± 1.0″.

**Table 1:** The three selected days are listed with indication of time, spectral line and seeing values. The seeing errors refer to the atmospheric fluctuations occurrence during the ~60 min needed for each observation.

Date	sp. line	Start Time (UT)	Seeing (″)	Date	sp. line	Start Time (UT)	Seeing (″)
07/06/2012	D2	09:42	1.9 ± 0.6	20/09/2012	D2	09:13	2.0 ± 0.6
	D2	10:53	1.8 ± 0.6		D2	10:17	1.7 ± 1.5
	D2	15:19	1.8 ± 0.6		D2	11:21	1.8 ± 0.8
	D2	18:02	1.8 ± 0.7		D2	12:25	1.5 ± 1.3
	D2	19:03	1.8 ± 0.9		D2	13:30	1.8 ± 1.0
25/05/2013	D1	12:22	1.9 ± 0.6		D2	14:35	2.0 ± 0.7
	D1	14:34	1.8 ± 0.7		D2	15:38	2.0 ± 0.9
	D1	15:39	1.3 ± 1.0		D2	16:43	1.8 ± 1.2
	D1	16:45	1.8 ± 0.7		D2	17:46	1.8 ± 0.8
	D1	17:53	1.8 ± 0.6				

In general, by looking at the 2D-pixel images – result of the reduction analysis – we do not see evident discrepancy in the size of Mercury disk obtained by the composition of the many slits of one image. Seeing values are derived by comparing the observed continuum image (reflected from the surface) with the theoretical image derived by the Hapke theory of surface reflectance. Table 1 shows that generally the seeing variability is very small, and anyway all of them superpose to each other within errors. The careful analysis of the continuum images (not shown) assures that good quality of data is provided. The images are derived in kRayleigh. Conversion to zenith column abundances is not applied in this context, since we are analyzing only morphological aspects.

**Figure 1:**
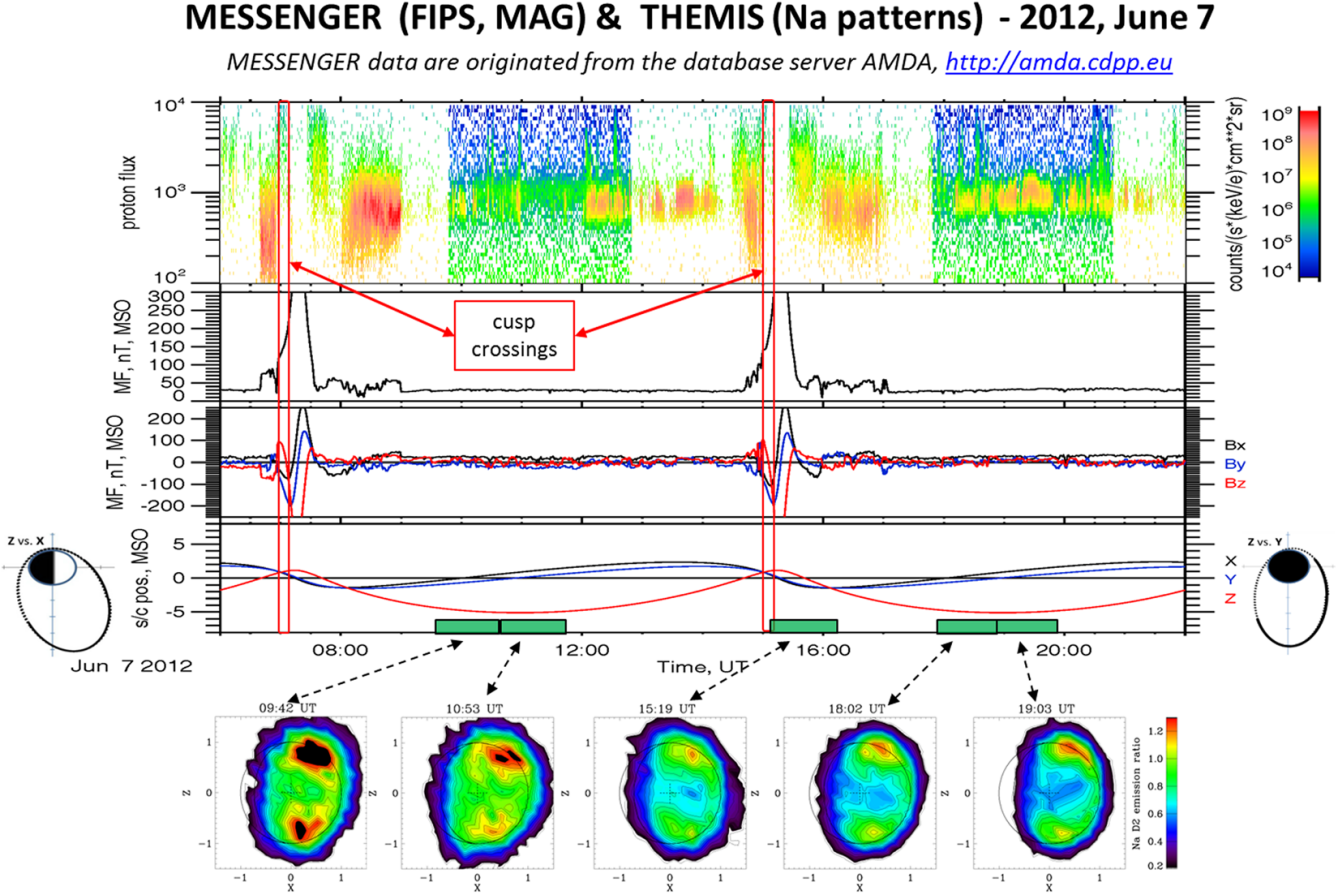
2012, June 7 time profiles. The upper four panels show the MESSENGER profiles vs. time of (from the top): FIPS proton spectrograms (the two cusp traversals are marked by red bars), MAG magnetic field (MF) magnitude, MAG MF components (MSO), s/c ephemeris components (MSO). At the two sides of the ephemeris plot, s/c anti-clockwise orbital paths are shown, respectively on the Z-X plane (left), and on the Z-Y plane (right). The bottom pictures are the 1-hour-based Na dayside patterns observed by THEMIS. The arrows point to the tick bars on the time scale, indicating the time intervals of each measurement. MESSENGER data are originated from the database server AMDA, http://amda.cdpp.eu/. The color scale to the right relates to an intensity index that is normalized respect do the average Na intensity profile vs. TAA, as derived by Leblanc and Johnson^5^.

The first mentioned representative case (2012, June 7, 06:00–22:00 UT) is shown in Fig. 1. Mercury was at TAA ~ 55°. The upper four panels show the MESSENGER profiles vs. time as described in the caption (FIPS; MAG, and ephemeris). Every time the S/C reaches the periapsis, the MF magnitude shows a steep increase every time the S/C crosses the magnetopause, as it enters/exits the planet magnetosphere, while in the solar wind the IMF magnitude profile is generally constant, at about 30 nT, indicating solar wind mostly undisturbed conditions. In fact, FIPS spectrograms show solar wind particles at about 1 keV. It should be noted that the IMF magnitude exceeds 25 nT, so that reconnection may occur continuously^10^, independently on the IMF components, that in this case are characterized by positive MF-X, and MF-Y and -Z randomly distributed around 0.

In these undisturbed conditions, the planet’s bow shock should stay at nominal distance from the planet (1.45 R_M_^23^), reconnection should occur continuously (IMF > 25 nT) and solar wind particles should regularly precipitate towards the surface through the cusps. Such plasma precipitation signatures are actually detected by FIPS when crossing the northern cusp, just at the S/C entrance inside the magnetosphere, in both the two traversals shown in the figure (UT about 6.45 and 14.45, see red bars). Actually, FIPS observation of precipitating particles is limited to the cusp traversal close to the polar regions, since this instrument is not able to measure precipitating protons across the dayside within 30° of the subsolar point (because MESSENGER traverses that region at too high of an altitude). It follows that any possible cusp shift at lower latitudes could not be monitored by MESSENGER.

The bottom pictures of Fig. 1 show the 1-hour Na emission images observed by THEMIS. The color scale shows the relative intensity with respect to average conditions, as described in the ‘Methods’ section, so that only relative variations are left. The images show two-peak patterns, above both polar regions. Although the intensity of the two peaks is fluctuating throughout the whole time period, the general configuration appears to be quite constant.

**Figure 2:**
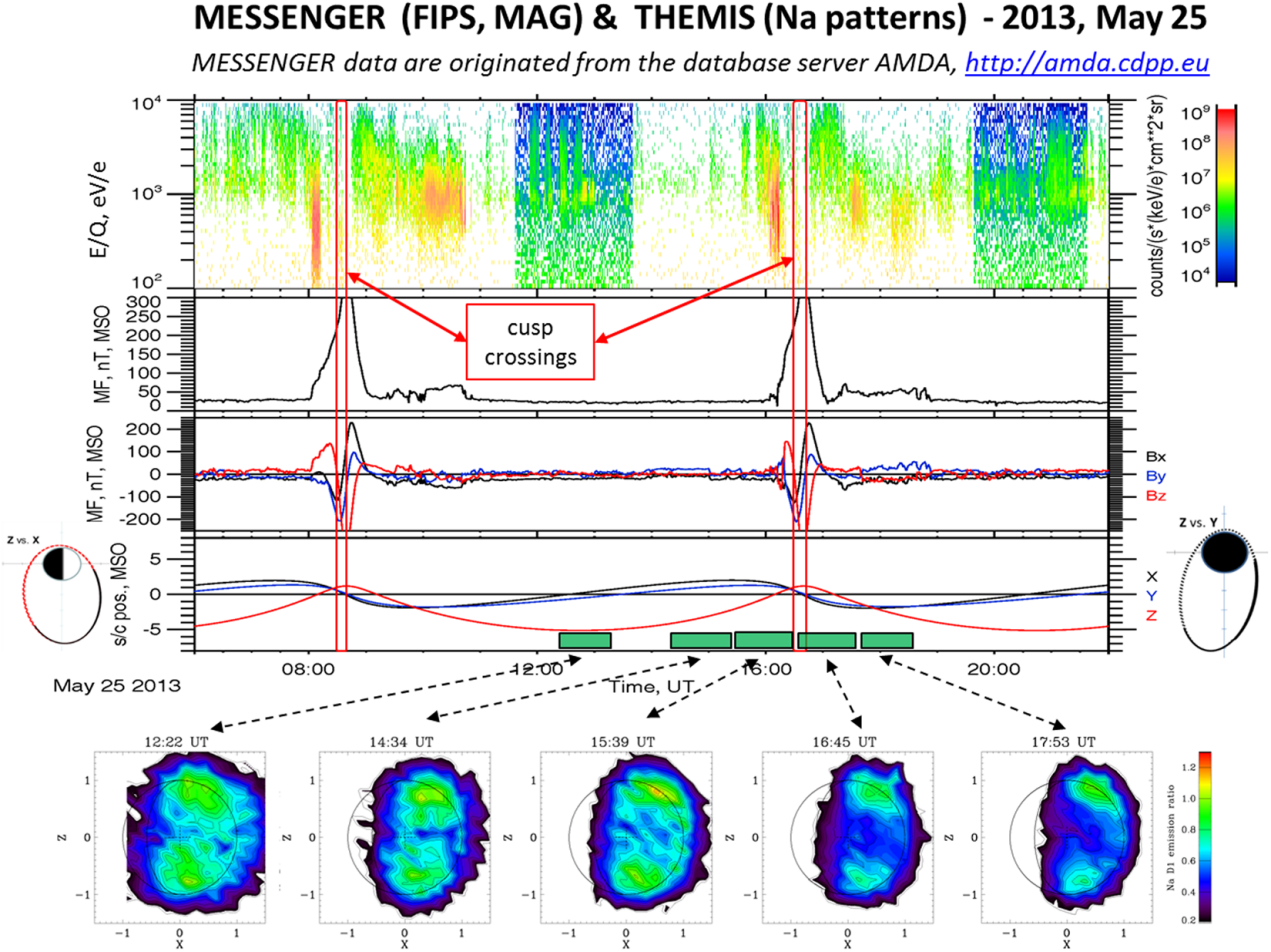
2013, May 25 time profiles. See Fig. 1 captions for description. MESSENGER data are originated from the database server AMDA, http://amda.cdpp.eu/.

The second selected case (2013, May 25, 06:00–22:00 UT) is shown in Fig. 2. The figure layout is the same as in Fig. 1. Again we notice from the MAG data two magnetosphere traversals; the IMF stays constantly around 25 nT. TAA is similar to previous case, ranging at ~57°. Again Na two-peak patterns are observed. The FIPS data show also in this case proton precipitation signatures throughout the cusp regions, actually traversed before the magnetosphere crossings (soon after 08:00 and after 16:00 UT, respectively, see the red bars).

**Figure 3:**
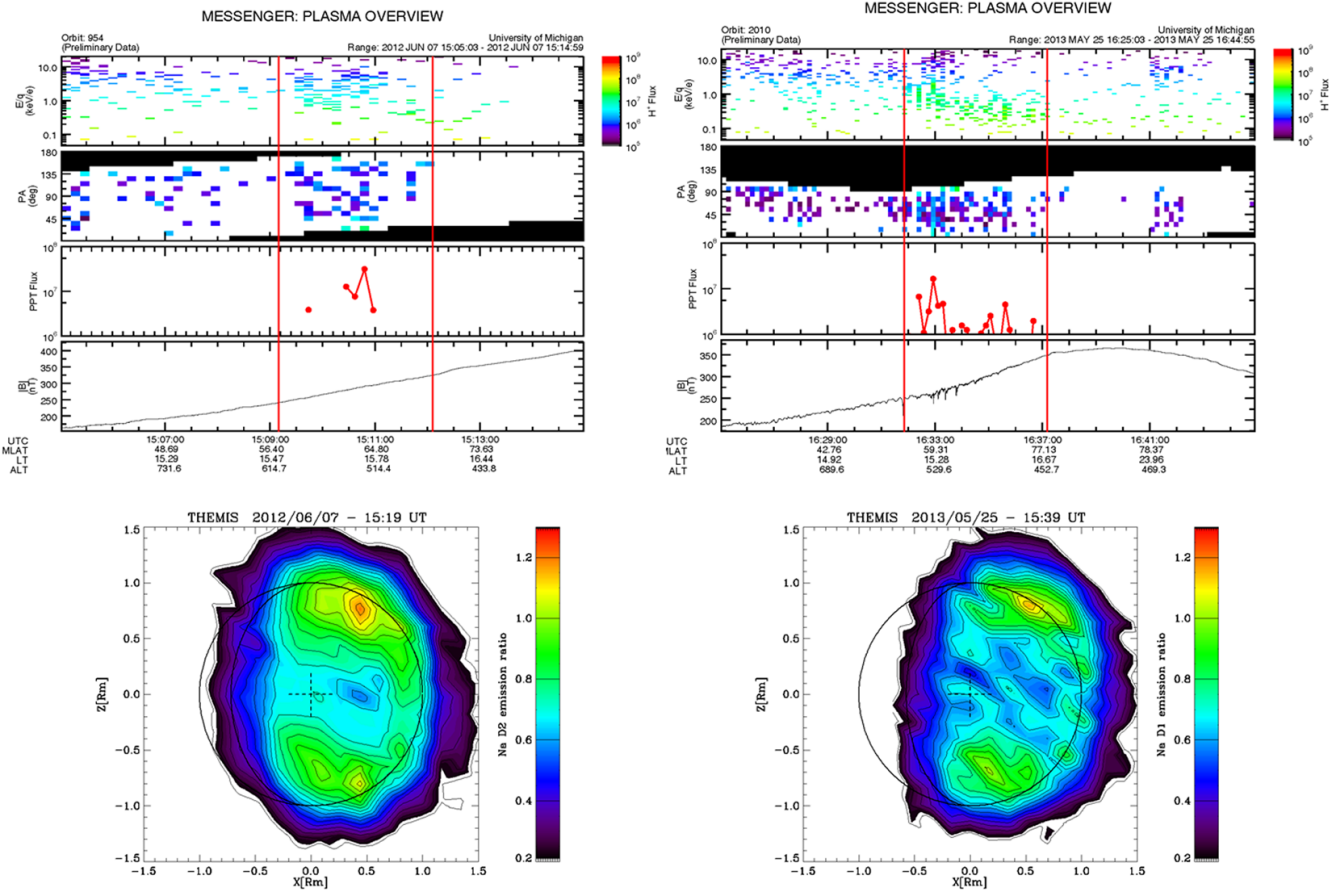
Top panels: 2012, June 7 15:05–15:15 (left), and 2013, May 25, 16:26–16:45 (right) time profiles. From the top: FIPS proton flux spectrograms, FIPS pitch angle respect to MF, FIPS downward flowing flux intensity, MF magnitude. The red lines delimitate the cusp crossing periods. The bottom panels show two hourly Na signal patterns: (left) 2012, June 7, 15:19–16:19, and (right) 2013, May 25, 15:39–16:39.

By assuming that magnetospheric regimes of reconnection and associated precipitation could influence the Na average emission in a specific period, we now focus, within both the selected events, on the specific moments when MESSENGER traverses the northern cusp region and almost simultaneously the Na emission is observed by THEMIS (Fig. 3). The left case relates with the 2012 June 7 event (FIPS and MAG plots: 15:05–15:15; THEMIS hourly observation: 15:19–16:19). The northern cusp traversal is indicated by the two vertical lines on the plot panels: it shows a typical energy trend versus time (from higher energy at lower latitudes to lower energies at higher latitudes), caused by the particle drift paths on Mercury’s dayside^11,36^. The pitch angle histogram (2nd from top) shows the FIPS field of view (FoV) in the frame of the magnetic field: the black regions are unobserved, the white are observed but zero flux, and the colored regions are where flux ≠0 is observed. As MESSENGER entered the cusp, FIPS could measure within 10° of the magnetic field direction. That increased to within 40° by the time that MESSENGER exited the cusp. The protons with pitch angles less than 90° are flowing downward (toward the surface) when they were measured by MESSENGER. In the 3rd panel from top, the flux of protons that should hit the surface (precipitate) is shown. Because in this case FIPS could not see all the way down to 0° pitch angle, the PPT flux computed here has to be taken as a lower limit (about 8 × 10^7^ cm^−2^ s^−1^, in this case). The pitch angle panels in the plots show that FIPS was able to view pitch angles from 0° to about 100°, so that the calculation of precipitation flux is quite reliable. The induced Na emission pattern (the one localized in the northern polar region) exhibits a relative intensity of the order of about 1.2, with respect to the nominal trend versus TAA^5^. The right case relates instead to the 2013 May 25 event (FIPS and MAG plots: 16:25–16:45; THEMIS hourly observation: 15:39–16:39). The two vertical lines identify the northern cusp traversal as observed by FIPS, showing again the typical energy versus time trend, from high to low energy. In this case the cusp precipitating proton fluxes are about 5 times lower than in the previous case.

The two cases here analyzed basically refer to mostly undisturbed solar wind conditions (occurring also in the other 7 sequences not described here). Hence, also by considering that the two events occur at the same TAA (55°and 57°),we may infer that two-peak Na emission pattern could be a proxy of the mostly undisturbed solar wind conditions (when IMF > 25 nT) at the Mercury location. In fact, even if we take into account that the Na images are responses obtained in 1 hour (hence, only roughly related to the impulsive plasma precipitation observed by MESSENGER), the fact that the IMF magnitude is constant throughout the whole period confirms the idea that proton precipitation through the cusps may occur regularly, being the reconnection process driven by IMF magnitude constant trend, and resulting in a constant trend of the Na emission. While Mercury’s Dungey cycle is of the order 2–3 min, it is unlikely that the cusp is completely reconfigured on that time scale at all times. Significant reconfiguration requires significant changes in the solar wind conditions. Moreover, the surface reaction to such precipitating particles occurs over a time scale much longer than 2–3 min, so that any possible intermittent pulse of particle precipitation could not affect the observed Na patterns.

**Figure 4:**
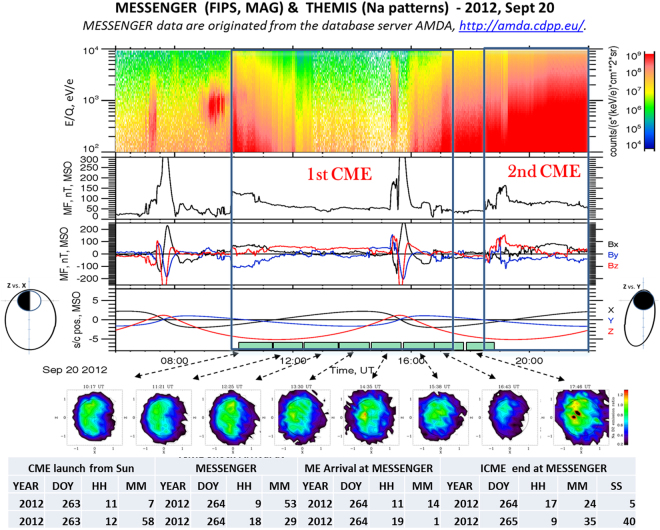
2012, Sept 20 time profiles. See Fig. 1 captions for description. MESSENGER data are originated from the database server AMDA, http://amda.cdpp.eu/. T the bottom, info on timing of two ICME transits identified by MESSENGER 36 is given.

Within our 10 selected data, we also measured a single sequence when a strong solar perturbation was hitting Mercury, as it was also detected by MESSENGER. In fact, Winslow *et al*.^36^ analyzed the MESSENGER magnetospheric data for discriminating the ICME transits at Mercury. They notice that, simultaneously with the THEMIS observations of Sept 20, 2012 a solar disturbance transited at Mercury: specifically, it was composed by two separate ICME-induced shock fronts, one just after the other. In Fig. 4, we show the MESSENGER and THEMIS data for this time period (TAA ~128°). At the bottom of Fig. 4, specific information on the solar disturbances are given, taken from^37^. The arrival of two ICMEs is recorded by MESSENGER: the first ICME transits at 09:53, and the second one at 18:29. Related FIPS spectrograms are mostly saturated, demonstrating the occurrence of Solar Energetic Particles (SEP) penetrating the instrument. The MAG data indicate two traversals of the magnetosphere (~06.00–09.00 UT and 15.00–17.00 UT). Moreover, at the time of the two ICME transits, two other abrupt jumps of the IMF magnitude are noticed (~10.00 and 18.30 UT). In the whole time period, the IMF magnitude reaches 150 nT max, and on average ranges around 60–80 nT, except for two time periods: a short period at 12:45–13:20 and in-between the end of the first ICME and the arrival of the second one (17:24–18:29). In both time periods, the IMF goes down to ~40 nT, much closer to the quiet time conditions.

The hourly Na images from THEMIS of that day are available for the whole period from 10:17 to 18:46. The first ICME transit starts before the first available Na image, whereas the second ICME transit starts within the last Na image. Actually, out of the selected data (seeing < = 2″), there is another Na image starting at 9.15, at the ICME start. Nevertheless, it is very similar to the next one, and in any case it does not provide any information on previous ICME conditions. By looking at the patterns sequence, first of all we notice that the Na emission is very diffused on the dayside, sometimes hardly showing two peaks very close to the equatorial region (1st and 2nd images), sometimes centered on the equatorial region (4th, 5th, 6th, and 8th images). The 3rd image, taken at 12:25–13:25 and corresponding to the first IMF decrease, shows a much more evident two-peak pattern. In the 7th image, at 16:43–17:43, only a very tenuous signal is recorded, just in the period when the first ICME transit vanishes, and before the second ICME arrival. In this time frame, MESSENGER was just exiting from the magnetosphere. The most intense and diffused pattern is observed in the 8th image at 17:46–18:46, just when the second ICME transit starts.

The events shown in Fig. 4 suggest that in condition of strongly enhanced IMF, like at the occurrence of ICME, the Na exospheric signal, induced by particle precipitation, is mainly much diffused in the equatorial region, though sometimes it exhibits two peaks pattern hardly visible and very close to the equator. Actually, out of the selected 10 sequences, a similar pattern of diffused emission is observed (but only a single image is available within our selection criteria) on May 20, 2013 (at 7:59 UT) during the transit of another ICME occurred between May 19 at 21:00 and May 20 at 20:48 UT. This lonely image confirms our results, obtained thanks to such a unique opportunity to show a whole time sequence of exospheric evolution during an ICME transit at Mercury.

## Discussion and Conclusions

Given the two different Na patterns shown in Figs 1, 2 and 4 and corresponding MF values, the IMF magnitude seems to be an indirect tracer of all particle precipitation conditions at Mercury. Unfortunately, direct measurement of the plasma thermal pressure is not available in the MESSENGER data. Anyway the low-β condition (β = (nkBT)/(B2/(2μ0)), quite nominal in the magnetosheath of Mercury, and generally in the whole inner heliosphere^28^, allows inferring that at least at a first order of approximation and within short time intervals, the IMF variations may be inversely related to β variations.

**Figure 5:**
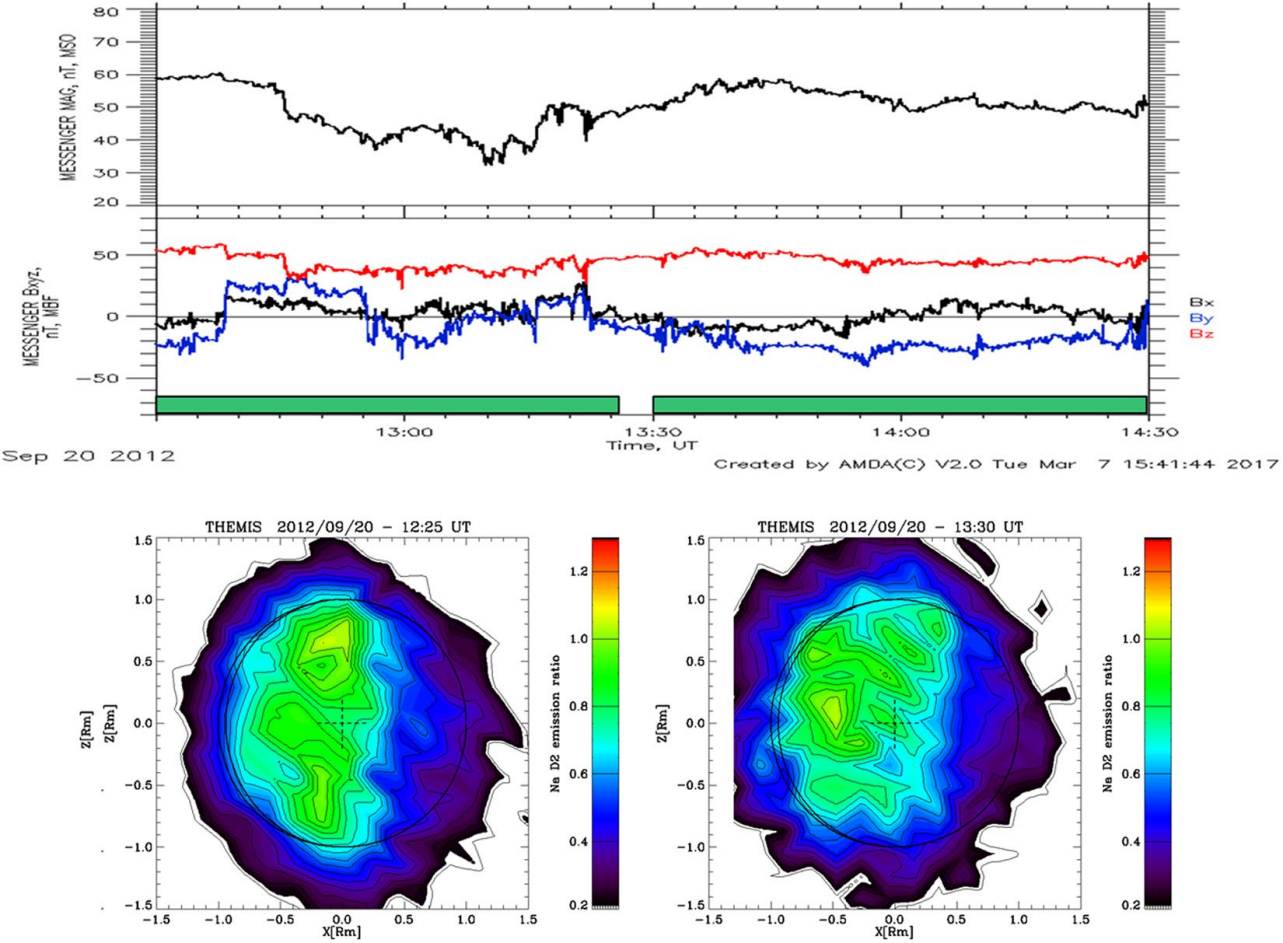
(Top) 2012, Sept 20. 12:30–14:30, time profile of MESSENGERE MAG MF magnitude and components; (bottom) THEMIS Na emission hourly patterns 12:25–13:25, and 13:30–14:30. The two thick bars along the time scale indicate the intervals of the two hourly patterns. MESSENGER data are originated from the database server AMDA, http://amda.cdpp.eu/.

As a matter of fact, Fig. 5 focus on the Sept 20 2012 Na emission during a IMF magnitude decrease and a subsequent increase, taken from Fig. 4 at 12:45–14:30: here a first double peak pattern, relative to open field lines precipitation time conditions (but a little more extended to lower latitudes) changes abruptly into an equatorial diffused pattern. Such change fits in the present scenario; in fact, in the occasion of any IMF decrease, the cusps are expected to move farther from the planet equator, and consequently the particles precipitation area should move towards the polar regions.

One hour later, when the IMF magnitude signal increases again, up to about 60 nT, a diffused Na pattern is observed. Such an abrupt pattern modification could be due to different particle precipitation regimes: the first linked to the cusp footprints (at mid-latitudes or below), the second driven by extreme compression of the magnetopause (Fig. 5, left and right panels, respectively). This last configuration allows the magnetosheath particles to precipitate anywhere on the dayside whenever the magnetopause is compressed down to or below an altitude of the order of the particle gyro-radii (hundreds of km).

In summary, the Na exospheric emission observed in the analyzed 10 sequences database (with average seeing < = 2) leads to two alternative scenarios of particle precipitations:

1. Open field lines plasma precipitation, which originates high-latitude two-peak Na emission. This is a frequent condition induced by significant reconnection rate at Mercury (low plasma β). At ICME transits, they cause thick, low-β plasma depletion layers and even higher reconnection rates, so that the cusps extend to lower latitudes, causing open field lines broad plasma precipitation areas^32^, with the two-peak Na patterns still existing, but hardly distinguishable.
2. Occasionally, at the ICME transit, the magnetopause itself approaches the planet surface. We see evidence that such a distance may be small; lower than particle gyroradii, i.e., a few hundred km, so that magnetosheath plasma may precipitate, independently from open field lines, on the planet’s dayside^31,34^. Actually, although increases in the planetary field from core induction offset, to some extent, the effects of compression, they do not rule out compression of the magnetopause to near the surface at low dayside latitudes (to within 0.02–0.13 mercury radii, 48–320 km, near the subsolar point and closer in the southern hemisphere)^32^. The hot proton distributions evident in the dayside magnetosheath in Fig. 4 extend to ~4 keV in the first crossing (07:07–07:23) and to almost to 10 keV in the second (15:18–15:32). Near the subsolar point these protons would have gyroradii of a few hundred km^11^, allowing them to impact the surface at low latitudes via gyration.
3. Particle precipitation is the major driver of Na surface release, so that the observation of Na emission evidence planetary space weather features at Mercury. This view was anticipated by Killen *et al*.^38^ that attributed the Na emission pattern variability to ion sputtering, acting where the solar wind precipitates, according to reconnection linked to IMF components. We have here proved that IMF components do not play a significant role with respect to the IMF magnitude itself, when IMF exceeds ~25 nT. The shown observations do not allow identification of the surface release process responsible for exospheric Na refilling. Anyway, considering that ion sputtering yields are generally not sufficient for sustaining the observed release, we expect that plasma impact on the surface would drive the enhanced diffusion process, which should be able to provide free Na atoms, then released in a short while through PSD process^15^. Such a link between plasma precipitation and Na release is here proved by observations for the first time.

These results prelude to a promising new scientific roadmap of the Earth-based Na exosphere monitoring as an actual proxy of solar wind status, at the Mercury position, in support of the understanding of the solar disturbances propagation in the Solar System. Actually, the use of *in-situ* observations (from space-borne satellites) in the inner heliosphere for predicting the ICME propagation has been the subject of several studies^39,40^ (and references therein), where the need of such information for properly deriving the basic ICME parameters is evidenced. The possibility to use Mercury exosphere as a constant ICME monitor would be of great profit for the modeling efforts in this crucial field. It follows that a continuous observation of the planet would be desirable. Actually, a frequent observation of the Na exospheric intensity and distribution at Mercury is already possible, together with the Sun ICME emission monitoring, by allocating time and resources to the solar telescopes presently available. In this way, Mercury could become a natural monitor of ICME propagation far from their generation sources on the Sun, as well from the Earth’s orbit.

## Methods

### THEMIS data analysis

VM performed a long Earth-based campaign of observations of the Na exosphere of Mercury in years 2007–2014 with the THEMIS solar telescope, located in Tenerife (Canary islands, Spain) by using MTR spectrograph and two independent cameras to investigate simultaneously small spectral regions around D1 and D2 emission lines (5896–5890 Å). The Mercury exosphere was automatically scanned over by a slit in the North-South direction and the image recomposed in a second step. Tip-tilt corrections at ~1 kHz were performed to assure perfect coverage of the planetary disk and to improve the quality of images. For each position of the slit, 20 exposures of 20 seconds were taken. Bias correction was applied and flat-field correction was performed by using the solar spectrum (taken in between usual Mercury observations throughout the day). Spectral calibration was performed by using solar spectrum too and by identifying telluric lines. Sky background was removed by using ‘line-free’ regions of the same spectra, and solar reflected spectrum too. Resulting exospheric emission line was then integrated and calibration in brightness was performed by using Hapke theory to model the solar light reflected from Mercury surface. Best fit of modeled Hapke reflected solar light with the observed one allowed us to derive exact position of the Mercury disk above the emission and estimate seeing values. More details of the analysis can be found in^3^. Finally, bi-dimensional map of the Na exospheric intensity emission, superposed on the disk of Mercury, are obtained. Such maps are then normalized over the average intensity emission expected at the TAAs of observations (as exposed in Fig. 3 in^5^) to evidence the relative intensity with respect to the average condition and exclude seasonal effects.

### FIPS data analysis

The computation of FIPS fluxes and pitch angle distributions from raw data is described in the documentation which accompanies the data in the Planetary Data System. FIPS proton precipitation flux was computed by propagating the observed pitch angle distribution (see Fig. 3, Panel 3 fm top) down to the surface assuming a radial magnetic field and conservation of the first adiabatic invariant. After correcting for the increasing flux due to converging field lines, the pitch angle distribution at the surface is Integrated over all pitch angles from 0 to 90° to give the precipitating flux. See^25^ for further details.

### Data Availability

The data that support the plots within this paper and other findings of this study are available from the corresponding author upon reasonable request. THEMIS data are online at: http://themis.iaps.inaf.it. FIPS data are available at AMDA (http://amda.cdpp.eu/) as well as at the Planetary Data System (PDS). MAG data are available at AMDA (http://amda.cdpp.eu/).
